# Mortality Outcomes in People with Lung Cancer with and without Type2 Diabetes: A Cohort Study in England

**DOI:** 10.2147/CLEP.S498368

**Published:** 2025-07-17

**Authors:** Eseosa Grace Igbinosa, Bodini Dharmasekara, Jennifer K Quint, Sanjay Popat, Krishnan Bhaskaran, Daniel Morganstein, Sarah Cook

**Affiliations:** 1 School of Public Health, Imperial College London, London, UK; 2 School of Computer Science and Mathematics, Keele University, Staffordshire, UK; 3 Lung Unit, The Royal Marsden NHS Foundation Trust, London, UK; 4 Department of Non-Communicable Disease Epidemiology, Faculty of Epidemiology and Population Health, London School of Hygiene & Tropical Medicine, London, UK; 5 Department of Endocrinology, Chelsea and Westminster Hospital NHS Foundation Trust, London, UK

**Keywords:** lung cancer, type 2 diabetes, mortality, cardiovascular mortality, respiratory mortality

## Abstract

**Introduction:**

The impact of type 2 diabetes (T2DM) on mortality following lung cancer diagnosis remains unclear, with conflicting evidence across studies. We aimed to assess differences in all-cause and cause-specific mortality between people with lung cancer with and without T2DM within a primary care population in England.

**Methods:**

The study population was 69,674 people with incident lung cancer within the Clinical Practice Research Datalink (CPRD) Aurum primary care database (2010–2022). The study exposure was T2DM at cancer diagnosis, and the outcomes were all-cause and cause-specific mortality (cancer, cardio-vascular, respiratory). Cox models were fitted for each outcome adjusting for age, gender, smoking status, body mass index, calendar year and socioeconomic status (Index of Multiple Deprivation).

**Results:**

After adjusting for age and gender, there was no evidence for a difference in all-cause mortality in people with T2DM compared with people without T2DM (IRR 0.98 95% CI 0.96, 1.01). After fully-adjusting for measured confounders, there was a small positive effect (IRR 1.07 95% CI 1.04, 1.09). After adjusting for age and gender, people with T2DM had lower rates of cancer-specific mortality compared to people without T2DM (IRR 0.96 95% CI 0.94, 0.98). However, after adjustment for all measured confounders there was a small positive association (IRR 1.05 95% CI 1.02, 1.07). In both age and gender adjusted and fully adjusted models people with T2DM had higher cardiovascular (fully adjusted HR 1.30 95% CI 1.15, 1.47) and respiratory disease mortality (fully adjusted HR 1.30 95% CI 1.15, 1.47).

**Conclusion:**

There was robust evidence that people with T2DM had higher cardiovascular and respiratory disease mortality following lung cancer diagnosis. The relationships between T2DM and all-cause and cancer-specific mortality were highly sensitive to adjustment for confounding. Differences in studies on approaches to confounding and levels of missing data may contribute to the mixed findings on this association in the literature.

## Plain Language Summary

Lung cancer and type 2 diabetes are both common conditions that have a big impact on health outcomes worldwide. There is some evidence that people with type 2 diabetes have a higher chance of dying if they develop certain cancers than people without type 2 diabetes. It is not clear if having type 2 diabetes affects people’s chances of dying if they develop lung cancer, or if any increased risk of death is due to lung cancer or other conditions such as heart disease. We used data from general practice records linked with hospital and death records from England to investigate whether people with diabetes were more or less likely to die following being diagnosed with lung cancer overall and from three specific causes of death: cancer, heart disease and lung disease.

People with type 2 diabetes were more likely to die from heart disease and lung-related illnesses after being diagnosed with lung cancer than people without type 2 diabetes. However, differences in dying overall and of dying from cancer were small and depended on how other factors, like age and smoking, were considered. These findings highlight the importance of managing heart and lung health in people living with type 2 diabetes who are diagnosed with lung cancer.

## Introduction

Cancer and Diabetes Mellitus (DM) are common diseases that contribute to high global morbidity and mortality. It is estimated that 20% of people with cancer have DM, mostly Type 2 DM (T2DM).^1^ Several epidemiological studies have suggested patients with T2DM may experience higher all-cause mortality following a diagnosis of cancer.^2–4^ It remains unclear if this increased mortality is driven by worse cancer outcomes or by an increased risk of death from other conditions such as cardiovascular disease. Some studies have reported worse cancer-specific mortality, notably in breast and colorectal cancer.^5^ However, there is potential bias in determining the cause of death, with evidence that in people with diabetes, diabetes is more frequently recorded on death certificates if someone dies of cardiovascular disease than if they die of cancer.^6^ Conversely, deaths in people with diabetes have a higher probability of being attributed to cardiovascular causes.^7^ Notably, there are conflicting results from studies assessing outcomes to cancer treatments in diabetes (reviewed in Joharatnam-Hogan et al 2023).^8^

Lung cancer, classified into Non-Small Cell Lung Cancer (NSCLC) and Small-Cell Lung Cancer (SCLC), is the third most diagnosed cancer in the UK accounting for 21% of all cancer-related mortality.^9^ Despite recent advances in diagnosis, staging and treatment, the 5-year survival rate for lung cancer remains unfavourable, at 19.4%.^10^

The impact of co-morbid T2DM on lung cancer survival remains unclear as the evidence to date has shown mixed results. Whilst several studies have shown worse overall survival,^11–18^ both following surgical resection,^11^ and in advanced disease,^14–17^ this is not universal and some studies have shown no difference^2^ or the opposite effect– that prevalent T2DM is associated with better survival from lung cancer.^19^,^20^ Studies to date have been limited by small sample sizes, often single centre, and have used varying methods to identify DM. In particular, a large proportion of studies have been conducted in East Asian populations, which may differ from Western populations in smoking rates, diet, exercise, insulin resistance levels, and biological lung cancer subtypes,^21^ all risk factors for and potential confounders of the relationship between lung cancer and T2DM and therefore not necessarily generalisable. Although some studies have suggested an increased risk of lung cancer in those with diabetes,^22^ more recent large studies, including those using Mendelian randomisation do not support an increased risk of lung cancer in those with diabetes.^23^,^24^ However, they are both common conditions, and numerous studies have shown that up to 20% of people with lung cancer have co-occurring DM.^1^ The impact of this is unclear. This study aimed to investigate whether T2DM is associated with differences in all-cause and cause-specific mortality in individuals diagnosed with lung cancer. We utilised a large Nationally representative database of primary care records linked with hospital records and Office for National Statistics (ONS) death registration data in England.

## Methods

### Data Source

Data came from the Clinical Practise Research Datalink (CPRD) Aurum May 2022 primary care Dataset.^25^ CPRD Aurum contains anonymised primary care data from more than 60 million patients and can be linked to secondary care and other health-related datasets,^26^ giving researchers a complete view of data regarding patient care, facilitating the conduct of essential studies to enhance patient care. A limitation for this study was that data on cancer-specific factors (stage, sub-type, treatment) are not included. The dataset was linked with the ONS^27^ and Index of Multiple Deprivation (IMD) data from 2022.^28^ ONS data included information on the official date and causes of death (using ICD-10 codes) of each patient, while patient-level IMD, which is a measure of relative deprivation for small areas (Lower Super Output Areas (LSOAs) was used as a proxy for socio-economic position. Linked pseudonymised data was provided for this study by CPRD. Data is linked by NHS Digital, the statutory trusted third party for linking data, using identifiable data held only by NHS Digital. Select general practices (GP) consent to this process at a practice level with individual patients having the right to opt-out.

### Study Population

We included people with a lung cancer diagnosis (measured by first mention of lung cancer within their primary care record) between 1st January 2010 and 1st January 2023 using SNOMED-CT codes entered by GPs during consultations.

**Figure 1:**
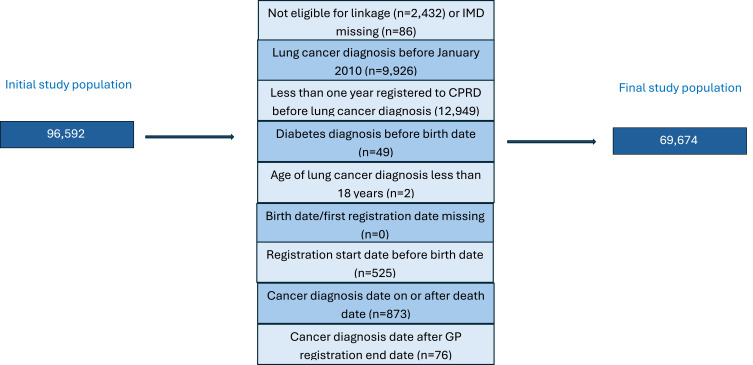
Exclusion criteria for patients included in the analysis, Reasons for ineligibility with the number of participants excluded.

People were included if they had a code for lung cancer recorded for the first time between 2010 and 2022, they were aged 18 or over at the time of lung cancer diagnosis, were eligible for data linkage with ONS data, were regular (not temporary patients) and had research acceptable data available from CPRD. People with less than 1 year of GP registration at the time of their first code for lung cancer were not included as this may reflect a historical diagnosis recorded post registration with a new GP Practice. Patients with inconsistent data, for example, date of death prior to date of birth were excluded. The number of people included in the study and reasons for exclusions are shown in Figure 1.

The code lists used for defining the study population, study exposures, outcomes and co-variates are available on Github.^29^

### Exposure Variable

The main exposure in this research work was the prevalence of T2DM at the time of lung cancer diagnosis. Patients having codes categorised as T2DM if they had a code recorded from codelist for prevalent diabetes codes (see codelist available at^29^) prior to their first code for lung cancer. Diabetes diagnosis after the first lung cancer was not included (whether related to cancer treatment or not).

The codelist for prevalent diabetes was derived by searching the CPRD browser (February 2022) using search terms related to diabetes and remerging with SNOMED Concept IDs to look for additional terms, which may have been missed. All codes included in the case definition were categorised by two coders including one clinician and consensus reached on what to include. Any codes categorised as T2DM, diabetes (type not specified) and diabetes-related (type not specified) and diabetes monitoring were included as prevalent T2DM on the basis that T2DM is the majority of diabetes. Among those classified as T2DM in the study, 83% had at least one code specific to T2DM within their primary care record. Codes which specified diabetes as Type 1 diabetes or something other than type 2, eg gestational diabetes, steroid induced diabetes were not included. As only diabetes diagnosis prior to the cancer diagnosis were considered, steroid-induced diabetes as a result of cancer treatment was not included.

### Outcomes

The primary outcome was all-cause mortality following lung cancer diagnosis (as measured by first code within primary care). Survival was calculated from the initial event (date of lung cancer diagnosis defined as first mention in primary care) to the final event (death or censoring (loss to follow-up due to switching to a non-CPRD registered practice or last collection date of the practice)). Patients with an ONS death date were determined as having died, while those without an ONS death date were assumed to be alive.

The secondary outcome was cause-specific mortality divided into cardiovascular, cancer-specific (any cancer) and respiratory causes as the underlying cause of death.

### Co-Variates

We accounted for several confounding variables that could potentially influence the relationship between lung cancer mortality and T2DM. These variables were selected based on their established associations with both T2DM and lung cancer outcomes and included: age, sex, IMD, calendar year of lung cancer diagnosis, smoking status, and body mass index (BMI).

Smoking status was categorized into three groups: current smokers, ex-smokers and never-smokers.

BMI was used as a categorical variable, we used the following classification categories from the World Health Organisation: Obese (>30.0), Overweight (25.0–29.9), Normal (18.5–24.9), and Underweight (<18.5). Sensitivity analysis was conducted comparing results using BMI as categorical with using BMI as a continuous variable. Most recent BMI and smoking status record prior to Lung Cancer diagnosis were defined using previously established codelists and algorithms.^30^ Since BMI and smoking status can change over time and more recent measurements prior to lung cancer diagnosis may be affected by reverse causality a sensitivity analysis was conducted excluding smoking and BMI records in the previous 12 months prior to lung cancer diagnosis.

Among people with T2DM the most recent HbA1c measurement (as mmol/mol) prior to lung cancer diagnosis was recorded as an indicator of diabetes control using categories (≤47.5; 47.5–69.4; 69.4–85.8; >85.8). HbA1c was only used for describing the study sample and was not included as a co-variate in the survival analysis. Codelists used to define co-variates are available within a Github repository.^29^

### Statistical Analysis

Continuous variables were presented using means and standard deviations, whereas categorical measures were reported using numbers and proportions.

We fitted separate Cox Regression Models for each outcome. Start of follow-up was the date of first code for lung cancer recorded within primary care records. End of follow-up was the earliest of the outcome (death or cause-specific death), death from other causes (for cause-specific mortality outcomes), end of GP registration or end of the study (1st January 2023).

All models were adjusted for age and gender. To understand the impact of adjusting for different confounders models were then adjusted for the remaining confounders (BMI, smoking status, year of diagnosis of lung cancer and IMD) one at a time in turn and then all together. The final models were compared to minimally adjusted models restricted to people with no missing data on any confounding variables (complete case analysis). To investigate the impact of missing data for BMI and smoking, age and gender-adjusted models were also fitted on the full data set and analyses adjusting for smoking and BMI individually were compared to models with complete data for those variables (ie, the model including adjustment for age, gender and smoking was compared to a model adjusted for age and gender among people with no missing data for smoking).

We tested the proportional hazards assumption using log–log plots of survival, Kaplan–Meier and predicted survival plots and Schoenfeld residuals. Where proportional hazards assumption was not met, data were stratified by cumulative follow-up time (<6 months; 1 year; more than 1 year).

All analyses were performed using R^31^ and Stata 18.^32^ R libraries used were “Survival”, “survminer” and ggplot2.

### Ethical Declaration

CPRD has NHS Health Research Authority (HRA) Research Ethics Committee (REC) approval to allow the collection and release of anonymised primary care data for observational research [NHS HRA REC reference number: 05/MRE04/87]. Each year CPRD obtains Section 251 regulatory support through the HRA Confidentiality Advisory Group (CAG), to enable patient identifiers, without accompanying clinical data, to flow from CPRD contributing GP practices in England to NHS Digital, for the purposes of data linkage [CAG reference number: 21/CAG/0008]. The protocol for this research was approved by CPRD’s Research Data Governance (RDG) Process (protocol number: 23–002741) and the approved protocol is available upon request. Linked pseudonymised data was provided for this study by CPRD. Data is linked by NHS Digital, the statutory trusted third party for linking data, using identifiable data held only by NHS Digital. Select general practices consent to this process at a practice level with individual patients having the right to opt-out.

## Results

### Study Population

The study population consisted of 69,674 eligible patients with lung cancer after applying specific exclusion criteria (Figure 1). Out of the final study population, 20.9% of people had T2DM.

Table 1 shows the baseline characteristics of the cohort by whether people had T2DM or not. People with T2DM were more likely to be male (T2DM: 58.1% vs non-T2DM: 51.9%) and diagnosed with cancer at an older age compared with people without T2DM (T2DM mean 74.4 (SD 9.2) vs non-T2DM: 71.9 SD (10.9)).

**Table 1:** Baseline Characteristics of Patients with and without Type 2 Diabetes Mellitus (T2DM) (N = 69,674)

Variable	T2DM (%)	No T2DM (%)
N	14,566 (100)	55,108 (100)
Gender		
Male	8455 (58.1)	28,578 (51.9)
Female	6111 (42.0)	26,530 (48.1)
Mean and SD Age of Lung Cancer Diagnosis	74.4 (9.2)	71.9 (10.9)
Median year of lung cancer diagnosis (IQR)	2017 (2013–2019)	2016 (2013–2019)
IMD quintile		
1 (least deprived)	2064 (14.2)	9193 (16.7)
2	2521 (17.3)	10,450 (19.0)
3	2646 (18.2)	10,470 (19.0)
4	3314 (22.8)	11,387 (20.7)
5 (most deprived)	4021 (27.6)	13,608 (24.7)
Smoking Status		
Never	1343 (9.2)	5374 (9.8)
Ex-smoker	4626 (31.8)	15,212 (27.7)
Current Smoker	8592 (59.0)	34,372 (62.5)
Missing^a^	5	150
BMI (Kg/m^2^)		
Underweight (<18.5)	440 (3.0)	3376 (6.4)
Normal (18.5 −25)	4017 (27.8)	22,367 (42.6)
Overweight (25 −30)	5111 (35.3)	17,484 (33.3)
Obese (>30)	4897 (33.9)	9304 (17.7)
Missing^a^	101	2577
HbA1c Level (mmol/mol)		
HbA1c ≤ 47.5	5486 (40.6)	25,048 (97.0)
47.5 <HbA1c ≤ 69.4	6565 (48.6)	759 (2.9)
69.4 < HbA1c ≤ 85.8	1054 (7.8)	12 (0.05)
HbA1c > 85.8	407 (3.0)	11 (0.04)
Missing^a^	1054	29,278

**Note**: ^a^Missing data values were excluded from statistical analysis.

**Abbreviations**: T2DM, Type 2 diabetes mellitus; BMI, Body mass index; SD, standard deviation; IQR, inter-quartile range; IMD, Index of multiple deprivation.

There was a higher proportion of people who were ex-smokers in the T2DM group although this difference was small (T2DM: 31.8% vs non-T2DM: 27.7%). Most people in the study sample, both with and without T2DM, were currently smoking (T2DM: 59.0% vs non-T2DM: 62.5%).

People with T2DM had a higher proportion of people with diabetes classified as BMI>30 (T2DM: 33.9% vs non-T2DM: 17.7%).

During the study period, there were 50,351 deaths (72.2% of the study cohort). The main cause of death was cancer (45,971 deaths; 91.3% of study deaths). The number of deaths due to CVD was 1469 (2.9% of study deaths) and due to respiratory disease was 1446 (2.9% of study deaths).

Follow-up time was 7798.2 person years for people without T2DM and 17,876.6 person years for people with T2DM. Absolute rates of all outcomes were higher in people with T2DM (Table 2).

**Table 2:** Rates of All-Cause and Cause-Specific Mortality Following Lung Cancer Diagnosis in People with and without Known Type 2 Diabetes (T2DM) at Time of Lung Cancer Diagnosis

		Number of Events	Rate (per 1000 Person Years) (95% CI)
All-cause mortality	People with T2DM	10,313	576.9 (565.9, 588.1)
	People without T2DM	40,038	513.4 (508.4, 518.5)
Cancer-specific mortality	People with T2DM	9213	515.4 (505.0, 526.0)
	People without T2DM	36,758	471.4 (466.6, 476.2)
Cardiovascular disease mortality	People with T2DM	376	21.0 (19.0, 23.3)
	People without T2DM	1093	14.0 (13.2, 14.9)
Respiratory disease mortality	People with T2DM	344	19.2 (17.3, 21.4)
	People without T2DM	1102	14.1 (13.3, 15.0)

**Abbreviations**: T2DM, Type 2 diabetes mellitus; CI, Confidence interval.

The hazard ratios for all-cause and cause-specific mortality following Lung cancer diagnosis among people with and without T2DM adjusting for confounders are shown in Table 3.

**Table 3:** Hazard Ratios for All-Cause and Cause-Specific Mortality Following Lung Cancer Diagnosis Among People with and without Type 2 Diabetes (T2DM)

Adjusted For:		All- Cause Mortality	Cancer-Specific Mortality	Cardiovascular Mortality	Respiratory Mortality
		IRR (95% CI)	IRR (95% CI)	IRR (95% CI)	IRR (95% CI)
Age + gender		0.98 (0.96, 1.01)	0.96 (0.94, 0.98)	1.26 (1.12, 1.42)	1.19 (1.05, 1.34)
+ calendar year of lung cancer diagnosis		1.04 (1.01, 1.06)	1.01 (0.99, 1.04)	1.33 (1.18, 1.50)	1.25 (1.11, 1.41)
+ IMD		0.98 (0.96, 1.00)	0.95 (0.93, 0.98)	1.24 (1.10, 1.40)	1.16 (1.02, 1.31)
Smoking Status (n=69,519)	Age + gender (no missing data on smoking status)	0.98 (0.96, 1.01)	0.96 (0.94, 0.98)	1.26 (1.12, 1.41)	1.19 (1.05, 1.34)
	+ smoking status	0.99 (0.96, 1.01)	0.96 (0.94, 0.98)	1.26 (1.12, 1.42)	1.19 (1.06, 1.35)
BMI (n=66,996)	Age + gender (no missing data on BMI)	1.00 (0.97, 1.02)	0.97 (0.95, 1.00)	1.26 (1.12, 1.42)	1.17 (1.04, 1.32)
	+BMI	1.03 (1.00, 1.05)	1.00 (0.98, 1.03)	1.25 (1.11, 1.41)	1.27 (1.12, 1.43)
Age + gender (no missing data on all variables) (n=66,968)		1.00 (0.97, 1.02)	0.97 (0.95, 1.00)	1.26 (1.12, 1.42)	1.16 (1.03, 1.32)
+ BMI +smoking status +IMD		1.02 (1.00, 1.04)	0.99 (0.97, 1.02)	1.24 (1.10, 1.40)	1.24 (1.09, 1.40)
+ BMI +smoking status +IMD + calendar year of lung cancer diagnosis		1.07 (1.04, 1.09)	1.05 (1.02, 1.07)	1.30 (1.15, 1.47)	1.30 (1.15, 1.47)

**Abbreviations**: T2DM, Type 2 diabetes mellitus; BMI, Body mass index; IMD, Index of multiple deprivation; IRR, Incidence rate ratio; CI, confidence interval.

### All-Cause Mortality

After adjusting for age and gender, there was no evidence for a difference in all-cause mortality in people with T2DM compared to people without T2DM (IRR 0.98 95% CI 0.96, 1.01). This fluctuated between remaining null and a small positive effect after adjustment for all confounders and restricting the model to people with no missing data on any adjusted confounders (IRR 1.07 95% CI 1.04, 1.09) (Table 3).

### Cancer-Specific Mortality

After adjusting for age and gender, people with T2DM had lower rates of cancer-specific mortality compared to people without T2DM (IRR 0.96 95% CI 0.94, 0.98). This fluctuated between a lower rate, no evidence of a difference and a small positive effect after adjustment for all confounders and restricting the model to people with no missing data on any adjusted confounders (IRR 1.05 95% CI 1.02, 1.07) (Table 3).

### Cardiovascular Mortality

After adjusting for age and gender, people with T2DM had higher rates of cardiovascular disease mortality compared with people without T2DM (IRR 1.26 95% CI 1.12, 1.42). This remained consistent after adjusting for different confounders and restricting to people with complete data on all confounders (IRR 1.30 95% CI 1.15, 1.47) (Table 3).

### Respiratory-Related Mortality

After adjusting for age and gender, people with T2DM had higher rates of respiratory disease mortality compared with people without T2DM (IRR 1.19 95% CI 1.05, 1.34). This remained consistent after adjusting for confounders and restricting to people with complete data on all confounders (1.30 95% CI 1.15, 1.47) (Table 3).

### Proportional Hazards Assumption

There was evidence that the proportional hazards assumption was not met for cardiovascular disease mortality (based on Schoenfeld residuals p = 0.007). When the analysis was stratified by cumulative follow-up time after adjusting for measured confounders the increased rate in CVD mortality in people with T2DM was not seen in the first 6 months of follow-up (IRR 0.52 (0.25, 1.06) but there was in first year of follow-up (IRR 1.35 95% CI 1.14, 1.59) (Table 4).

**Table 4:** Hazard Ratios for CVD-Specific Mortality Following Lung Cancer Diagnosis Among People with and without Type 2 Diabetes (T2DM) Stratified by Cumulative Follow-up Time*

N=66,968	CVD Mortality	
Follow Up Time	Age + Gender	All Measured Confounders**
	IRR (95% CI)	IRR (95% CI)
<6 months	0.54 (0.27, 1.10)	0.52 (0.25, 1.06)
<1 year	1.35 (1.14, 1.59)	1.40 (1.18, 1.66)
Total sample	1.26 (1.12, 1.42)	1.30 (1.15, 1.47)

**Notes**: *Evidence against proportional hazards assumption from Schoenfeld residuals (p=0.007). **Age + gender + BMI +smoking status +IMD + calendar year of lung cancer diagnosis.

**Abbreviations**: T2DM, Type 2 diabetes mellitus; CVD, cardiovascular disease; BMI, Body mass index; IMD, Index of multiple deprivation; IRR, Incidence rate ratio; CI, confidence interval.

There was no evidence that the proportional hazards assumption was not met for the other three outcomes, therefore stratified results are not presented for these outcomes.

### Sensitivity Analysis

Sensitivity analyses excluding records for smoking and BMI, which were in the 12 months prior to lung cancer diagnosis (Supplementary Table 1) and comparing adjustment for BMI as a categorical variable and a continuous variable (Supplementary Table 2) produced results consistent with the main analysis.

## Discussion

Here, we investigated how prevalent T2DM at the time of lung cancer diagnosis was associated with all-cause and cause-specific mortality. There was strong evidence that people with T2DM had 30% higher CVD and respiratory disease mortality following lung cancer diagnosis. The relationship between T2DM and all-cause and cancer-specific mortality following lung cancer diagnosis was smaller and dependent on confounder adjustment. Differences in studies on approaches to confounding and levels of missing data may contribute to the mixed findings on this association in the literature. The increased risk of respiratory and cardiovascular mortality in those with diabetes may reflect known pathological alterations in type 2 diabetes, including vascular disease, increased inflammation and increased risk of infection. Alternatively, it is possible that this reflects altered treatment of underlying co-morbidities in the face of cancer treatment.^24^

### Comparison with Previous Literature

Our findings are not inconsistent with previous literature, which has shown mixed results with regard to the relationship between diabetes and all-cause mortality following lung cancer diagnosis.^2^,^11–17^,^19^,^20^ Our findings with regard to cause-specific mortality are consistent with existing literature that people with diabetes in general have an increased risk of dying from cardiovascular and respiratory causes.^33^

A previous meta-analysis has indicated that the relationship between diabetes and lung cancer survival may vary among various cancer subtypes and treatments, with surgically treated non-small cell lung cancer (NSCLC) showing the most pronounced association between diabetes and overall survival (Hazard Ratio: 1.71, 95% Confidence Interval: 0.94–3.08, p = 0.07).^34^ Here we have not stratified by cancer subtype or treatment and are not able to extrapolate our findings by these factors. However, we have utilised a large, multi-ethnic cohort, and used primary care coding of diabetes to determine cases, and national death certification data to determine cause of death, enabling a comparison of cause-specific mortality. This differs from most prior studies that have examined overall mortality, and our finding of increased respiratory and cardiovascular mortality, but not cancer-specific mortality may have implications of strategies to improve outcomes, suggesting a need to focus on cardiovascular health after lung cancer treatment.

### Strengths and Limitations

Here, we utilized a large nationally representative database linked to Office for National Statistics Death data. The study included a large sample size of close to 70,000 patients with lung cancer. The outcome was obtained from Office for National Statistics death records using underlying cause of death for cause-specific mortality analyses, however, there still remains the possibility of misclassification. We also did not account for multi-factorial cause of death using the underlying cause of death only. Misclassification for the exposure is a potential concern in our study,^35^ as we defined T2DM based on the presence of SNOMED CT codes for diabetes within primary care health records including codes for undefined DM diagnoses. This might have impacted the specificity of our study for T2DM as some patients with other types of diabetes may have been misclassified, potentially leading to biased estimates of association. Efforts to refine the coding criteria for diabetes diagnoses could help address this issue and enhance the accuracy of our results.^36^ Likewise, it is possible that some individuals with lung cancer do not have this coded on the primary care record, and linkage to other data sources such as Hospital Episode Data or National Cancer Registry data would lead to more complete data.

Our study leveraged the CPRD Aurum database, which utilizes routinely collected health record data not originally collected for research purposes. As a result, measurement and recording errors, as well as clinician coding variability, may have influenced our findings. For example, confounding factors such as smoking and body mass index were taken from the last recording within primary care records and therefore may not have accurately reflected status at the time of lung cancer diagnosis in all people. Furthermore, only smoking status was available and not data on the amount smoked such as pack-years. Importantly, certain crucial details such as cancer stage, subtype, and details of cancer treatment are not available within primary care data. We also were not able to adjust for all potential confounders, for example, data on air pollution and occupational exposure and environmental exposures were not available, and there may be residual confounding by unmeasured factors. We used the first lung cancer code as a surrogate for the date of cancer diagnosis and the accuracy of the initial diagnosis date might not be uniform across all cases. Primary care records are also not completely sensitive for capturing lung cancer diagnoses with a previous study using CPRD GOLD dating estimating sensitivity at approximately 65%.^37^ This may have led to a bias if the probability of being recorded within primary care is related to diabetes and mortality, although we are not aware of a reason why this should be the case. Notably, lung cancer is renowned for being diagnosed at an advanced stage,^38^ which introduces limitations in using the follow-up time from cancer diagnosis as a reliable indicator of cancer stage. It is also important to consider people with T2DM may have a higher probability of having their cancer detected and therefore to be diagnosed at an earlier stage given they are in more frequent contact with health care services,^39^,^40^ especially for smokers. It is important to acknowledge without accurate data on cancer stage, variations in mortality rates according to cancer stage and subtype could not have been accounted for and residual or unmeasured confounding may be important factors for explaining our results. Further research linking primary care data to other data sources such as cancer registry data, which include data on cancer staging and sub-type would strengthen the evidence base for this question.

As the study conditioned on lung cancer status, collider bias could have impacted our results. However, whilst a number of cancers have been shown to occur at increased frequency in those with diabetes, suggesting an aetiological link, this has not clearly been shown with lung cancer^41–44^ although it is a possibility. In the absence of a clear aetiological link it is therefore less likely that there is collider bias of the association with mortality following lung cancer diagnosis.

### Future Work

While this study has shed light on the intricate relationship between diabetes and cause-specific survival outcomes in patients with lung cancer, there remain several avenues for further research.

The current study primarily focused on mortality in all types of lung cancer. Future research could delve into more specific subtypes and stage of lung cancer, as the impact of diabetes on survival might vary depending on these factors. Stratifying the analysis by cancer stage and subtype could uncover nuanced associations that might not be apparent in overall survival analyses. Further research is needed to investigate whether there are disparities in outcomes based on sociodemographic variables such as ethnicity, geographic location, and healthcare access.

Given recent suggestions of metformin’s potential impact on survival in diabetic patients with non-small cell lung cancer, further research could explore the effects of specific diabetes medications on survival outcomes and its interaction with biological factors. Investigating potential synergies between diabetes treatments and lung cancer therapies could offer novel therapeutic avenues. Further investigation of diabetes drugs associated with reduced cardiovascular events, such as SGLT-2 inhibitors and GLP-1 analogues will also be of interest.

This study lays the groundwork for future investigations aimed at unravelling the complex relationship between diabetes and cause-specific survival outcomes in patients with lung cancer. In particular, our study findings highlight the need for strategies to improve survival for people with diabetes following cancer diagnosis to include a focus on addressing CVD risk factors given the increased risk of CVD mortality in this group.

## Conclusion

Among people with lung cancer identified through their primary care records, cancer-specific mortality was lower in people with prevalent T2DM at the time of their lung cancer diagnosis compared to people without T2DM but this was explained by adjusting for measured confounders. Cardiovascular and respiratory mortality among people with lung cancer was higher in people with prevalent T2DM.

## Data Availability

Data are available on request from the CPRD. Their provision requires the purchase of a license, and this license does not permit the authors to make them publicly available to all. This work used data from the version collected in May 2022 and have clearly specified the data selected within each Methods section. To allow identical data to be obtained by others, via the purchase of a license, the code lists will be provided upon request. Licenses are available from the CPRD (http://www.cprd.com): The Clinical Practice Research Datalink Group, The Medicines and Healthcare products Regulatory Agency, 10 South Colonnade, Canary Wharf, London E14 4PU.

## Abbreviations

BMI, Body Mass Index; CAG, Confidentiality Advisory Group; CI, Confidence Interval; CPRD, Clinical Practice Research Datalink; CVD, Cardiovascular Disease; DM, Diabetes Mellitus; GP, General Practice; HES, Hospital Episode Statistics; HRA, Health Research Authority; ICD-10, International Classification of Diseases −10; IMD, Index of multiple deprivation; IQR, Inter-quartile range; IRR, Incidence rate ratio; LSOA, Lower super output areas; NHS, National Health Service; NSCLC, Non-Small Cell Lung Cancer; ONS, Office for National Statistics; OR, Odds ratio; REC, Research Ethics Committee; SCLC, Small cell lung cancer; SD, Standard deviation; T2DM, Type 2 Diabetes.
